# Influence of health food literacy on willingness to pay for healthier foods: focus on food insecurity

**DOI:** 10.1186/s12939-024-02135-1

**Published:** 2024-04-22

**Authors:** Su-Jung Nam, Jaehye Suk

**Affiliations:** 1 Department of Consumer Sciences, Convergence Program for Social Innovation, College of Social Sciences, Sungkyunkwan University, Seoul, Republic of Korea; 2 Convergence Program for Social Innovation, College of Social Sciences, Sungkyunkwan University, Seoul, Republic of Korea

**Keywords:** Healthier food literacy, Food security household, Food insecurity household, Food insecurity

## Abstract

**Background:**

The repercussions of food insecurity are widely recognized to negatively impact overall health and are influenced by a complex interplay of physiological, psychological, social, and environmental factors.

**Methods:**

This study examined the disparities in food consumption and literacy between among food security households and food insecurity households using data from the Korea Rural Economic Institute’s 2022 Consumer Behavior Survey for Food, which involved 3,321 respondents.

**Results:**

Food security households had a greater understanding of and better attitude toward healthier food choices than food insecurity households. Economic ability was identified as having the most significant association with food purchasing behavior, with food security households spending more on average than food insecurity households. Structural equation modeling demonstrated the association of knowledge and attitude with dietary implementation and underscored the significance of consumer literacy as a factor related to willingness to pay for healthier foods.

**Conclusions:**

This study underscores the intertwined relationships among financial capacity, knowledge, and health-conscious dietary choices. It also suggests the need for targeted interventions addressing economic and educational gaps to foster healthier food consumption patterns across different socioeconomic contexts.

## Background

In developed nations such as the United Kingdom and the United States, a discernible price differential exists between more healthful dietary options and their less nutritionally robust alternatives [1–4]. Survey data from the United Kingdom reveal that a significant number of households face difficulties in purchasing nutritious foods because of cost [5]. This economic burden contributes significantly to food insecurity [6]. Food insecurity pertains to not only the availability of food but also the affordability of healthier choices [7–9]. The repercussions of food insecurity are widely recognized to negatively impact overall health in developed nations and are influenced by a complex interplay of physiological, psychological, social, and environmental factors. Developing effective public health strategies and interventions that address inequalities in food consumption requires a deeper understanding of how individuals experience food insecurity and the precise mechanisms that affect food consumption.

Amid the increasing global emphasis on healthier foods, the importance of consumers’ literacy regarding their dietary choices has become even more crucial. Consumer literacy, particularly regarding dietary choices, is not only about imparting knowledge but also about equipping individuals with the ability to make informed choices that suit their needs and situations [10, 11]. When consumers are well-informed and literate, they can mitigate the challenges associated with food insecurity. Conversely, a lack of such literacy could deepen the crisis of food insecurity [12, 13]. Interestingly, consumers with literacy clearly understand the nutritional value and health benefits of various foods, and such wisdom often stems from education, campaigns, or personal interests [14, 15]. However, the application of this literacy can vary significantly, depending on the socioeconomic context.

In terms of socioeconomic dynamics, households with adequate resources often prioritize nutritional value over cost. By contrast, economically strained households may need to expand their understanding of healthy foods and their capacity to integrate them into their daily diets because of financial limitations [16–18]. This duality raises the compelling hypothesis that the impact of consumer literacy initiatives may vary based on household economic profiles.

Recognizing these subtleties requires a departure from universal strategies and underscores the necessity for a context-specific examination of food consumption behaviors. In analyzing these dynamics, adopting a more nuanced approach becomes imperative to acknowledge the diversity in household composition, characteristics, and available resources. In the current study, we acknowledge that distinctions in consumers’ food literacy serve as a pivotal foundation for fostering behaviors associated with the consumption of healthful foods. Accordingly, this study aims to meticulously examine food literacy, particularly its intersection with individuals’ food security. The overarching goal is to discern and illuminate the challenges inherent in food consumption within demographics characterized by low-income strata and, consequently, to propose well-founded solutions. This study may be significant in the broader academic landscape as it can provide indispensable foundational data. Such data play a pivotal role in informing the development and formulation of nuanced policies and educational initiatives that are precisely tailored to address the identified challenges and enhance food literacy, particularly within socioeconomically disadvantaged sectors of the population.

Motivated by these complexities, the current study investigates these relationships in depth. We aim to scrutinize their manifestation across diverse household types, effectively going beyond simplistic or binary categorizations of “literate” and “non-literate” households. Specifically, we define a suite of pivotal research questions that seek to probe variations in demographic characteristics, food consumption behavior, and levels of consumer literacy across different households. First, what are the demographic characteristics of different household types? Second, what are the food consumption behaviors of different household types? Third, how does healthier food literacy differ by household type? Fourth, what is the relationship between factors related to healthier food literacy? Fifth, does the relationship between factors related to healthier food literacy vary according to household?

By addressing these research questions, this study enriches the current understanding of the factors that shape healthier food consumption and illuminates the intricate interrelationships among these factors.

## Theoretical framework

### Food security

Food security has been increasingly recognized as a multifaceted concept that moves beyond the sole consideration of food availability to capture the broader dimensions of food access, utilization, and stability over time. According to the Food and Agriculture Organization [19], food security exists when “all people, at all times, have physical and economic access to sufficient, safe and nutritious food that meets their dietary needs and food preferences for an active and healthy life.” Thus, food security forms the cornerstone of public health, socioeconomic stability, and human dignity and serves as an essential measure of a community’s health and economic well-being [20].

The converse of food security, that is, food insecurity, refers to the inadequate and unstable access to nutritious food. Its prevalence poses a pervasive threat to individuals and communities and contributes to a range of detrimental health and socioeconomic outcomes [21]. Food insecurity can lead to malnutrition, exacerbate mental distress, and adversely affect quality of life [22]. This insecurity extends beyond individual health implications and resonates with broader societal concerns. The profound link between food insecurity and detrimental health consequences has been extensively documented in scholarly research, which underscores its importance as a pivotal policy issue [22, 23].

A fundamental element of food security is the knowledge of food, that is, understanding what to eat, when to eat, and how to source and prepare nutritious food. Such knowledge is particularly critical for low-income groups that may be increasingly vulnerable to food insecurity in the absence of adequate information. Food knowledge is integral to maintaining dietary diversity and nutritional adequacy, especially in contexts constrained by limited resources [24].

Previous studies have suggested a correlation between food insecurity and poor diet quality, which is often attributed to a lack of knowledge about healthier food choices [25, 26]. In particular, diets lacking essential nutrients have been identified as significant contributors to chronic conditions such as obesity and diabetes [27, 28]. These findings emphasize that food insecurity can lead to a range of health issues because of malnutrition as well as the absence of information or understanding of healthy dietary choices.

### Food literacy

Food literacy involves the practical aspects of maintaining a healthy diet and encompasses skills such as planning, managing, selecting, preparing, and consuming food [29]. This concept has evolved into a comprehensive framework to delineate the essential knowledge, attitudes, and behaviors required to adhere to a healthy diet in accordance with nutrition guidelines [29]. Adults with higher levels of food literacy tend to be more informed about nutrition guidelines, exhibit better dietary quality, and demonstrate more positive food-related behaviors than those with lower levels of food literacy [30, 31]. Individuals with enhanced food literacy demonstrate increased self-control and reduced impulsiveness in their eating habits. They tend to incorporate more fruits and vegetables into their diet and exhibit superior abilities in meal planning, food and beverage selection, and food preparation [30–34].

Knowledge of the benefits of healthier foods is an integral part of healthy food literacy. Previous studies have indicated that as individuals become more informed about food, they make better nutritional choices [35, 36]. Therefore, an enhanced comprehension of the advantages of nutritious foods, reflected by factors such as traditional food certification, the Korean industrial standard mark for processed food, hazard analysis critical control point, country of origin, certified organic status, geographic indication, traceability, good agricultural practices, and animal welfare certification, is likely to be closely associated with healthier food behavior. Building on this premise, this study formulates the following hypothesis:

#### H1. The knowledge of healthier food is associated with healthier food behavior

Moreover, attitudes toward healthier food, encompassing personal feelings, values, and motivations, exert a significant influence on food choices and behaviors. A positive attitude toward healthier food can encourage the regular consumption of fruits and vegetables and thus foster a balanced diet [37, 38]. This positive inclination toward healthier food options aligns with a better understanding of their nutritional value [39]. A heightened positive attitude toward healthier food may be closely linked to healthier food behavior, exemplified by factors such as traditional food certification, the Korean industrial standard mark for processed food, hazard analysis critical control point, country of origin, certified organic status, geographic indication, traceability, good agricultural practices, and animal welfare certification. Therefore, we hypothesize the following:

#### H2. The attitude toward healthier food is associated with healthier food behavior

The relationship between knowledge, attitudes, and healthier food behavior is multifaceted. Although knowledge does not always translate into behavior, research has highlighted the positive association between these factors. Greater knowledge of and positive attitudes toward healthier food are associated with a higher prevalence of healthier food behaviors [40, 41]. Previous research has suggested that individuals who are informed about healthier foods and maintain a positive attitude are more likely to invest in safer food options even if they are more expensive [42]. Based on this premise, the following hypotheses are proposed:

#### H3: The knowledge of healthier food is associated with the intention to pay a higher price for healthier food options

#### H4: The attitude toward healthier food is associated with the intention to pay a higher price for healthier food options

Finally, previous studies have provided evidence of a strong relationship between healthier food consumption behavior and willingness to pay more for food safety. This relationship is attributed to the increased awareness of the health benefits and potential risks associated with food choices [43–45]. Hence, we propose the following hypothesis:

#### H5: Healthier food behavior is associated with the intention to pay a higher price for healthier food options

## Methods

### Data

This study utilized data derived from the 2022 Consumer Behavior Survey for Food (CBSF) conducted by the Korea Rural Economic Institute (KREI). To ascertain food purchasing behavior and changes in consumer preferences in Korea, the KREI conducts the annual CBSF targeting Korean grocery shoppers aged 19–75 years in their households. The 2022 CBSF was conducted through face-to-face interviews from June 10 to August 21, 2022. In this study, the household list from the Statistics Korea Census was used for sample extraction. The samples were chosen using stratified sampling, with 16 metropolitan cities as the strata. Additionally, samples were sequentially extracted for the survey units and households within each stratum using stratified probability sampling, with the extraction probability being proportional to the total number of households in the survey unit. A total of 3,321 respondents were included in the analysis.

### Measures

Table 1 lists the measurements used in this study. The validity and reliability of all measurements have been confirmed in previous studies [46–49], with a particular focus on Cronbach’s $\alpha$ for items related to attitude that necessitate internal consistency. Households responding with “agree” or “strongly agree” to the question, “Did you have sufficient economic resources to purchase an adequate quantity and variety of food for your family in the last 12 months?” were categorized as **food security households**. By contrast, households that responded with “disagree” or “strongly disagree” were classified as **food insecurity households**. Those that selected “average” were placed in the **food marginal group**.

**Knowledge** was rated as “1” if well-known and “0” otherwise; then, the scores for 10 healthier food labeling items were summed. **Attitude** was defined as a consumer’s belief, affect, and behavioral tendency toward healthier food consumption [49]. It was measured using a 5-point Likert scale ranging from “not at all” to “very positive.” **Behavior** was rated as “0” to indicate the absence of any purchases of healthy food items in the last year and “1” to indicate the purchase of at least one healthy food item during that period. The rating scale was applied to the questions related to the purchase experience of nine measured items, and the scores were then summed. Finally, **intention to pay a higher price for healthier food** was measured using a 5-point Likert scale ranging from “not at all” to “very positive.”

**Table 1:** Measurement

	Definition		
**Demographics**			
Gender	Biological sex		
Age	Length of time in years that a person has lived		
Education	Level of schooling from which one graduated		
Occupation	The primary type of employment one is currently engaged in for livelihood		
Marital status	Whether one is currently in a married relationship		
Number of household members	The total number of family members currently residing together		
Monthly household income	The aggregate total of the monthly earnings of all household members		
	**Items**	**Source**	**Cronbach’s α**
**Food literacy**			
Knowledge	How much do you know about each of the following items? traditional food certification; Korean industrial standard mark for processed food; hazard analysis critical control point; country of origin; certified organic; geographic indication; traceability; good agricultural practices; genetically modified organismanimal welfare certification	Choi et al. (2010)	
Attitude	1. I tend to consider calories and nutritional content when I eat food. 2. I try to eat the five basic food groups in every meal for nutritional balance. 3. I eat a variety of foods for proper nutrition. 4. I eat lots of vegetables, fruits, and whole grains.	Song and Yoo (2008)	0.702
Behavior	Have you purchased foods with the following indicators within the past year? traditional food certification; Korean industrial standard mark for processed food; hazard analysis critical control point; country of origin; certified organic; geographic indication; traceability; good agricultural practices; animal welfare certification	Choi et al. (2010)	
**Intention to pay a higher price for healthier food**	Are you willing to pay a higher price for healthier food?		

### Analysis

Chi-square tests and t-tests were employed to assess whether the respondents’ characteristics, food consumption, knowledge, attitude, behavior, and intention to pay a higher price for healthier food were related to household type. Additionally, a structural equation model (SEM) was used to assess the path relationships on healthier food literacy. SEM estimates were used to evaluate hypotheses H1–H5. Hypothesis testing was conducted at a significance level of 0.05. All statistical analyses were performed using R version 4.1.0.

## Results

### Demographic characteristics by household type

Table 2 presents the demographic characteristics based on household type. Significant differences were observed among household types in terms of gender, age, education, occupation, marital status, number of household members, and monthly household income.

The food security and food marginal households mainly comprised individuals aged 50–59 years, making up 32.9% and 32.7% of the groups, respectively. Meanwhile, individuals aged 60–69 years were most prevalent in the food insecure households, representing 32.3% of the population. High school graduates represented 47.9%, 46.8%, and 50.4% of the respondents in the food security households, 46.8% in food marginal households, and 50.4% in food insecurity households, respectively. In addition, relatively high proportions of college graduates were observed in the food security and food marginal households, at 39.6% and 36.9%, respectively, while the food insecurity households had a notably higher percentage of middle school graduates at 25.2%.

Service worker was the most prevalent occupation in all three groups, although with the proportions of office workers and housewives were also relatively high. The proportion of married individuals was notably high in all three groups. Single-person households accounted for 34.1%, 32.5%, and 23.3% of the food insecurity households, food marginal households, and food security households, respectively. Finally, the distribution of monthly household income was relatively even in the food security and food marginal households. As for the food insecurity households, 23.0% and 23.9% reported monthly household incomes of 1.01 to 2 million KRW and 2.01 to 3 million KRW, respectively, indicating a higher percentage of low-income households.

**Table 2:** Demographic characteristics according to household type

Category	Total		Food Security Household		Food Marginal Household		Food Insecurity Household		χ^2^	*p*
	n	%	n	%	n	%	n	%		
Total	3321	-	1761	53.0	1334	40.2	226	6.8		
Gender									12.021	.003
Male	368	11.1	164	9.3	176	13.2	28	12.4		
Female	2953	88.9	1597	90.7	1158	86.8	198	87.6		
Age									57.645	.000
19–29	82	2.5	42	2.4	36	2.7	4	1.8		
30–39	347	10.4	203	11.5	122	9.2	22	9.7		
40–49	743	22.4	435	24.7	274	20.5	34	15.0		
50–59	1072	32.3	580	32.9	436	32.7	56	24.8		
60–69	789	23.8	383	21.7	333	25.0	73	32.3		
70–74	288	8.7	118	6.7	133	10.0	37	16.4		
Education									67.997	.000
No Education	53	1.6	18	1.0	26	2.0	9	4.0		
Middle school graduate	435	13.1	191	10.8	187	14.0	57	25.2		
High school graduate	1582	47.6	844	47.9	624	46.8	114	50.4		
College graduate	1235	37.2	698	39.6	492	36.9	45	19.9		
Post-graduate	16	0.5	10	0.6	5	0.4	1	0.4		
Occupation									53.613	.000
Administrator	28	0.8	20	1.1	7	0.5	1	0.4		
Professional Office worker	64 685	1.9 20.6	42 404	2.4 22.9	21 254	1.5 19.0	1 27	0.4 17.3		
Service worker Agriculture and fisheries	1185 258	35.7 7.8	621 125	35.3 7.1	480 109	36.0 8.2	84 24	37.1 10.6		
Skilled worker Housewife	330 744	9.9 22.4	173 365	9.8 20.7	136 315	10.2 23.6	21 64	9.3 28.3		
Others	27	0.8	11	0.6	12	0.9	4	1.8		
Marital status									41.106	.000
Married Not Married	2358 963	71.0 29.0	1331 430	75.6 24.4	889 445	66.6 33.4	138 88	61.1 38.9		
Number of household members									49.039	.000
1	922	27.8	411	23.3	434	32.5	77	34.1		
2	1215	36.6	669	38.0	453	34.0	93	41.2		
3	690	20.8	410	23.3	249	18.7	31	13.7		
4	447	13.5	247	14.0	176	13.2	24	10.6		
5	42	1.3	21	1.2	20	1.5	1	0.4		
6	5	0.2	3	0.2	2	0.2	0	0		
Monthly household income (in 10,000 KRW)^a^									98.152	.000
$\le$100	140	4.2	53	3.0	69	5.2	18	8.0		
101–200 201–300 301–400 401–500 501–600 601–700	439 691 599 510 505 252	13.2 20.8 18.0 15.4 15.2 7.6	184 344 315 292 318 140	10.4 19.5 17.9 16.6 18.1 8.1	203 293 244 187 175 102	15.2 22.0 18.3 14.0 13.1 87.71	52 54 40 31 12 10	23.0 23.9 17.7 13.7 5.3 4.4		
> 700	185	5.6	115	6.5	61	4.6	9	4.0		

*Notes*: ^a^1 USD = 1,303.43 KRW (as of July 10, 2023)

### Food consumption based on household type

Table 3 presents food consumption based on household type. Initially, it reveals that the frequency of food purchases per week was 1.87 for food marginal households and 1.60 for food insecurity households, with a statistically significant difference between the two groups. Furthermore, the average amounts spent on food purchases were KRW 64,231.12, KRW 63,221.89, and KRW 59,092.92 for the food security, food marginal, and food insecurity households, with the differences not being statistically significant. Finally, regarding food expenditure in 2022 relative to that in 2021, food security households reported a decrease (1.9%), no changes (56.8%), and an increase (41.3%) in food expenditure. The food marginal households saw a decrease (2.5%), no change (63.0%), and an increase (34.4%). The food insecurity households experienced a decrease (2.2%), no change (64.6%), and an increase (33.1%). The differences between the three groups were not statistically significant.

**Table 3:** Food consumption according to household type

	Total		Food Security Household		Food Marginal Household		Food Insecurity Household		*F/*χ^2^	*p*
	Means/n	SD/%	Means/n	SD/%	Means/n	SD/%	Means/n	SD/%		
Frequency of food purchases per week	1.84	1.51	1.85^ab^	1.50	1.87^b^	1.54	1.60^a^	1.42	3.211	.040
Average amount spent on food per purchases (in KRW)^a^	63,476.06	42215.37	64,231.12	42700.52	63221.89	42271.02	59,092.92	37732.33	1.525	.218
Food expenditure in 2022 vs. 2021									18.121	.001
Decrease	73	2.2	34	1.9	34	2.5	5	2.2		
No change	1987	59.8	1000	56.8	841	63.0	146	64.6		
Increase	1261	37.9	727	41.3	459	34.4	75	33.1		

*Notes*: ^a^1 USD = 1,303.43 KRW (as of July 10, 2023). ^a, b^ Significant difference in post-hoc Scheffé test at alpha = 0.05

### Food literacy based on household type

Table 4 shows the differences in healthier food literacy by household type. Statistically significant differences were noted in knowledge, attitude, behavior, and intention to pay a higher price for healthier food, and the scores of the food security households were higher than those of the food marginal and food insecurity households.

**Table 4:** Healthier food literacy according to household type

	Total			Food Security Household		Food Marginal Household		Food Insecurity Household		*F*	*p*
	M	SD	Range	M	SD	M	SD	M	SD		
Knowledge	3.75	3.28	0–10	4.20^c^	3.35	3.41^b^	3.19	2.26^a^	2.51	10.450	0.000
Attitude	3.45	0.54	1–5	3.56^c^	0.51	3.36^b^	0.53	3.13^a^	.57	103.8	0.000
Behavior	5.62	3.27	0–10	6.03^b^	3.22	5.08^a^	3.32	4.66^a^	2.92	19.09	0.000
Intention to pay higher price for healthier food	3.47	0.57	1–5	3.53^b^	0.56	3.42^a^	.57	3.37^a^	.58	17.46	0.000

*Note*: ^a, b^ Significant difference in post-hoc Scheffé test and Games–Howell test at alpha = 0.05

### Structural equation model of healthier food literacy

Table 5 shows the results of the SEM of healthier food literacy. The structural model accounted for 17.3% of the variance in preventive behaviors (adj. R^2^ = 0.173). The estimated structural model exhibited a good fit with χ^2^/df = 39.023 (*p* =.000), NFI = 0.923, CFI = 0.923, and RMSEA = 0.017. Knowledge was significantly associated with behavior (β = 0.385, *p* <.001) and intention to pay a higher price for healthier food (β = 0.096, *p* <.001). In addition, attitude was significantly associated with behavior (β = 0.110, *p* <.001) and intention to pay a higher price for healthier food (β = 0.126, *p* <.001). Meanwhile, behavior was significantly associated with intention to pay a higher price for healthier food (β = 0.130, *p* <.001).

**Table 5:** Estimation results of the structural equation model

		Estimate	SE	t	*p*
H1	Knowledge → Behavior	0.384	0.385	16.985	^***^
H2	Attitude → Behavior	0.661	0.110	4.828	^***^
H3	Knowledge → Intention to pay a higher price for healthier food	0.016	0.096	4.840	^***^
H4	Attitude → Intention to pay a higher price for healthier food	0.131	0.126	7.279	^***^
H5	Behavior → Intention to pay a higher price for healthier food	0.022	0.130	4.962	^***^

*Notes*: SE, standard estimation; t, t-value; p, p-value;^***^p <.001

### Multigroup analysis based on household type

Table 6 presents the results of the multigroup analysis comparing household types. In the analysis of food security households, the path coefficient from attitude to behavior was the only one that was not statistically significant. Within food marginal households, the path coefficient from attitude to the intention to pay a higher price for healthier food was the only one that was not statistically significant. For the food insecurity households, only the path coefficients from knowledge to behavior and from attitude to behavior were statistically significant. A comparison of the three groups also revealed statistically significant path coefficients for attitude → behavior, knowledge → intention to pay a higher price for healthier food, and attitude →intention to pay a higher price for healthier food. The last column in Table 6 displays the p-values derived by testing the equality of path coefficients from the three distinct SEM models.

**Table 6:** Multigroup analysis based on household type

					Food Security Household					Food Marginal Household					Food Insecurity Household				Δχ^2^	*p*
					Estimates	SE	t	*p*		Estimates	SE	t	*p*		Estimates	SE	t	*p*		
H1		Knowledge → Behavior			0.354	0.365	12.043	^***^		0.398	0.387	10.536	^***^		0.385	0.339	3.309	^***^	0.889	0.641
H2		Attitude → Behavior			0.223	0.035	1.167	0.243		0.924	0.150	4.093	^***^		1.493	0.296	2.888	0.004	7.585	0.023
H3		Knowledge → Intention to pay a higher price for healthier food			0.017	0.101	3.816	^***^		0.019	0.108	3.420	^***^		− 0.032	− 0.136	-1.716	0.086	6.117	0.047
H4		Attitude → Intention to pay a higher price for healthier food			0.174	0.161	6.886	^***^		0.052	0.049	1.762	0.078		0.031	0.030	0.394	0.693	11.025	0.004
H5		Behavior → Intention to pay a higher price for healthier food			0.013	0.073	2.145	0.032		0.034	0.199	4.669	^***^		0.046	0.225	1.829	0.067	5.790	0.055

*Notes*: SE, standard estimation; t, t-value; *p*, *p*-value; ^***^p <.001

## Discussion

In this comprehensive study, we meticulously examined the intricate interplay between food consumption patterns, spending behaviors, and consumer literacy in making healthier food choices within a diverse range of households. The core focus of our research is to uncover the disparities that arise between households endowed with ample economic resources to afford a diverse array of foods and those grappling with economic constraints that limit their options.

The analysis of household spending on food revealed no significant difference in the average amount spent on food purchases by food security, food marginal, and food insecurity households. This finding challenges the notion that financial capability alone dictates food procurement and consumption patterns. Instead, it suggests that while the frequency of food purchases per week varies, with food marginal households shopping more frequently than food insecurity households, the actual expenditure is consistent across different levels of food security. These findings align with those of several previous studies, underscoring the association of economic capacity with dietary habits and decisions [50, 51]. This relationship indicates that economic access and food affordability can significantly shape a household’s nutritional status and health outcomes.

When analyzing consumer literacy regarding healthier food alternatives, we noted significant disparities across households with divergent economic capabilities. Food security households scored notably higher in terms of knowledge about, attitudes toward, and behaviors associated with healthier food. These households also demonstrated a stronger willingness to pay higher prices for healthier food options. This observation resonates with prior studies underscoring the importantance of economic accessibility and affordability in making healthier food choices [52]. Furthermore, this evidence suggests that an increased willingness and ability to pay for healthier food are often associated with improved nutritional status and health outcomes.

Our application of an SEM further illuminated the interplay between knowledge, attitudes, and behavior. It revealed that knowledge and attitudes were significantly associated with dietary behaviors and that these factors, in turn, were significantly associated with the propensity to pay more for healthy food, a form of preventive behavior. This crucial insight emphasizes the role of consumer literacy as a significant factor associated with preventive health behavior and thus corroborates the conclusions of previous research [53–55].

Interestingly, a common pattern emerged across all economic strata. Regardless of economic status, all households demonstrated that knowledge was significantly associated with behavior and attitudes that profoundly shaped preventive behavior. These findings indicate that even in the face of economic challenges, knowledge and attitudes remain instrumental in determining dietary behavior and the willingness to invest in healthier food options [48–50]. Moreover, the financial capacity of households, although essential, is not the only driver of healthy dietary choices.

A noteworthy aspect of our study revolves around the varying associations of knowledge and behavior across different economic strata. We observed that the relationship between knowledge and behavior was not significantly different across food security and food insecurity households. This outcome suggests that the relationship between knowledge and behavior is consistent regardless of the household’s food security status, indicating the universally important role of knowledge in promoting behavioral outcomes related to healthier food choices. While the estimates of the path coefficients from knowledge to behavior were positive for both the food security and food insecurity households, the lack of a significant difference suggests that interventions aimed at increasing knowledge may be similarly effective for both groups. This indication highlights the potential for knowledge-based public health strategies to have a broad impact on varying levels of household food security. Intriguingly, knowledge was significantly associated with behavior in the food security households and food insecurity households.

Expanding our investigation, we examined the relationship between attitudes toward healthy foods and household purchasing behavior [56]. Notably, we found distinct patterns across different economic strata. In the food security households, knowledge and attitudes were significantly associated with the willingness to pay a premium for healthier food options. Intriguingly, our study identified another crucial factor beyond knowledge and prior purchasing experience. Nutritional content, caloric intake, and overall nutritional balance are critical aspects associated with purchasing decisions. This nuanced insight underscores the need for educational initiatives that focus on holistic nutritional education.

The results also highlight the need for educational programs that align attitudes with behavior, particularly within food security households. Emphasizing nutritional education that equips individuals with the ability to understand, interpret, and apply nutritional information to their daily food choices can bridge the gap between intention and action. Moreover, facilitating a deeper comprehension of the intrinsic link between holistic nutritional intake and long-term health benefits can enhance the likelihood of individuals consistently making health-conscious choices. Within food insecurity households, the connection between attitudes and purchasing behavior had a different trajectory. Our findings indicate that while attitudes are significantly linked to the decision to choose healthier options, they are not necessarily significantly associated with the willingness to pay more for such choices.

This study reveals that economic constraints pose a significant barrier to the acquisition of healthy foods for food insecurity households. Additionally, a lack of food literacy emerges as an impediment to the adoption of healthy eating habits. These findings are consistent with those of prior research [57–58] indicating a higher prevalence of obesity in demographic groups characterized by low household income. Within households facing economic limitations, a deficiency in food literacy increases the likelihood of opting for fast food [59], carbonated beverages [60], and high-fat foods [61] that provide immediate satisfaction, rather than selecting healthier alternatives. The consumption of these foods inevitably increases the risk of obesity and chronic diseases. Consequently, these dietary patterns are likely to adversely affect the health of food insecurity households. Therefore, creating targeted educational programs that focus on delivering accurate and relevant information regarding food choices may be beneficial. By effectively disseminating information on the nutritional value, health benefits, and practical aspects of healthier food choices, policies can ensure that households are equipped with the necessary tools to navigate the complex landscape of food options. This approach not only empowers them to make informed decisions but also opens pathways toward adopting healthier dietary practices.

Multifaceted policy interventions are required to address this challenge. Policies can create an environment in which healthier choices are financially viable for food insecurity households by subsidizing healthier food options and implementing incentive programs to mitigate economic barriers. Policy responses to food insecurity should strive to provide healthy and nutritious meals for at-risk populations. Additionally, they should encompass fundamental measures that address the systemic factors that impose competing demands on household finances. In Korea, government nutrition assistance programs, such as the congregate meal program and home-delivered meal services, primarily focus on older adults and individuals with disabilities. The primary foundations of food assistance in Korea consist of two national programs and temporary food support provided by local authorities [48, 62]. Currently, adults facing food insecurity in Korea lack dedicated and sustained food aid packages that consistently deliver adequate and nutritious meals in the long term [56]. Although income support programs extend supplemental cash benefits to financially strained households, they fail to address the simultaneous demands for nutrition and healthcare faced by marginalized individuals and households. Therefore, policy reforms should consider the interconnected dynamics of food consumption associated with food insecurity. Comprehensive interventions are essential to address the systemic factors contributing to food insecurity and to promote healthy food consumption among low-income adults and their families.

## Conclusions

This study elucidates the nuanced dynamics of food consumption patterns and consumer literacy across economically diverse households. The findings not only underscore the importance of food security considerations in food choice but also highlight the crucial role of knowledge and attitudes. This multifaceted understanding is pivotal for crafting effective strategies and interventions aimed at promoting healthier dietary habits across a wide range of socioeconomic contexts.

Nevertheless, this study has several limitations that need to be addressed in future work. SEM was employed to assess the relationship between the variables and test hypotheses H1–H5, particularly examining the relationship between retrospective and economic factors and the intention to consume healthy food. However, several potential endogeneity issues may exist, and they may include reverse causality, unobserved variables, and concurrency/common root causes. Hence, the results of this study are unavoidably confined to exploring potential associations or correlations between variables rather than making assertions about causal relationships. Accordingly, future research must undertake experimental studies to establish causal relationships between variables or utilize longitudinal data that demonstrate a clear temporal sequence and the ability to manipulate variables.

## Data Availability

The datasets generated and/or analyzed in the current study are available in the Korea Rural Economic Institute repository. [https://www.krei.re.kr/foodSurvey/index.do]

## Abbreviations

**CBSF**: Consumer Behavior Survey for Food
**KREI**: Korea Rural Economic Institute
**SEM**: Structural Equation Model
